# Elements in Serum, Muscle, Liver, and Kidney of Rabbits Fed Macroalgae-Supplemented Diets

**DOI:** 10.3390/md22060263

**Published:** 2024-06-07

**Authors:** Sabela Al-Soufi, Marta Miranda, Javier García, Antonio Muíños, Eugenio Cegarra, Nuria Nicodemus, Carlos Herrero-Latorre, Marta López-Alonso

**Affiliations:** 1 Departamento de Patoloxía Animal, Facultade de Veterinaria, Campus Terra, Universidade de Santiago de Compostela, 27002 Lugo, Spain; sabelaalsoufi.novo@usc.es (S.A.-S.); marta.lopez.alonso@usc.es (M.L.-A.); 2 Departamento de Anatomía, Produción Animal e Ciencias Clínicas Veterinarias, Facultade de Veterinaria, Campus Terra, Universidade de Santiago de Compostela, 27002 Lugo, Spain; 3 Departamento de Producción Agraria, Escuela Técnica Superior de Ingeniería Agronómica, Agroalimentaria y de Biosistemas, Universidad Politécnica de Madrid, 28040 Madrid, Spain; javier.garcia@upm.es (J.G.); nuria.nicodemus@upm.es (N.N.); 4 Porto Muíños S.L., 15185 Cerceda, Spain; antonio@portomuinos.com; 5 De Heus Nutrición Animal, 15004 A Coruña, Spain; ecegarra@deheus.com; 6 Departamento de Química Analítica, Nutrición e Bromatoloxía, Facultade de Ciencias, Campus Terra, Universidade de Santiago de Compostela, 27002 Lugo, Spain; carlos.herrero@usc.es

**Keywords:** rabbit, meat, essential and toxic elements, iodine, arsenic, macroalgae

## Abstract

The addition of marine macroalgae to animal feed has garnered interest due to the demonstrated benefits of gut health in many livestock species. Most macroalgae have a higher mineral content than terrestrial vegetables, making them an attractive, sustainable source of minerals. However, some macroalgae contain elevated concentrations of iodine and arsenic, which may be transferred to the meat of livestock fed with macroalgae. This study evaluated the mineral profile of rabbit serum, muscle, liver, and kidney of rabbits fed diets supplemented with different marine macroalgae, with the goal of improving post-weaning gut health and reducing reliance on antibiotics. We found increased deposition of iodine in muscle, liver, and kidney due to macroalgae supplementation, which is particularly promising for regions with low iodine endemicity. Higher, though relatively low arsenic concentrations, compared to those in other animal meats and food sources, were also detected in the muscle, liver, and kidney of macroalgae-fed rabbits. The absence of apparent interactions with other micronutrients, particularly selenium, suggests that the inclusion of macroalgae in rabbit diets will not affect the overall mineral content. Enhanced bioavailability of elements such as phosphorus and iron may provide additional benefits, potentially reducing the need for mineral supplementation.

## 1. Introduction

The rabbit meat industry is facing a critical period despite the meat being highly valued in many Mediterranean countries for its quality and nutritive properties [1,2]. Consumption of rabbit meat has declined in recent years due to the perception of rabbits as pets and a lack of availability of products adapted to modern consumption [3,4]. In addition, the industry is grappling with a serious challenge related to increasing post-weaning rabbit mortalities [5], mainly due to the complexity of maintaining gut health without antibiotics, recently restricted by the EU in animal production (Regulation (EC) No. 1831/2003). 

The inclusion of macroalgae in animal feed has become a very attractive strategy for improving the gut health of livestock because of the demonstrated benefits of these products, such as prebiotics, antioxidants, and immune modulators [6]. Macroalgae are also good sources of dietary fiber, high-quality protein, vitamins, polyunsaturated fatty acids, and polyphenolic compounds [7]. The high mineral content of macroalgae (8–40% DM of ash), exceeding that of any terrestrial vegetable [8], is particularly noteworthy. This feature makes macroalgae a great source of indispensable minerals for maintaining the gut health of animals, as is the case of iodine, zinc, or selenium, which are required in order to maintain gut health functionality as antioxidants, microbiome regulators, or immune modulators [9]. Moreover, some elements like iodine, iron, or zinc have been demonstrated to be more bioavailable in macroalgae than in other feed sources [10,11,12,13]. These characteristics could be exploited in animal feed production to provide an alternative, sustainable source of some minerals to meet recommended daily intakes [9,14]. However, mineral concentrations vary greatly among macroalgal species and in relation to environmental factors [15]. Macroalgae supplementation of animal feed requires fine-tuning [16]; therefore, a better understanding is required regarding the transfer of different elements to meat and other animal products intended for human consumption [16]. Among the essential trace elements in macroalgae, iodine has received the most attention because it can reach very high concentrations in some brown macroalgae [15] and is easily transferred to animal products (particularly milk and eggs, but also meat) when added to livestock feed [13,17]. Iodine supplementation via macroalgae has actually received much attention as a means of addressing iodine deficiencies in human populations [13], provided the toxic limits of this element are not exceeded [18]. The arsenic content has also garnered considerable interest as this potentially toxic element is found in high concentrations in some macroalgae [19], although it is generally of organic nature and of low toxicity [20]. By contrast, the high contents of other macroelements, such as sulfur and magnesium, in some macroalgae species [16], and the potential interactions between some elements—e.g., iodine and arsenic with selenium [21,22,23] and between sulfur and selenium [24,25,26]—have scarcely been studied in animals fed algae. Such elements warrant attention for both animal health and the residues in animal products.

The objective of the present study was to assess the mineral profile (including macrominerals, trace elements, and toxic elements) in the serum, muscle, liver, and kidney of rabbits fed experimental diets supplemented with four different marine macroalgae products as a strategy to reduce reliance on antibiotics by enhancing the gut health of post-weaning rabbits. Our hypothesis is that essential trace elements (especially iodine) may be transferred to the tissues, positively impacting human nutrition, while there will be no relevant accumulation of toxic elements (primarily arsenic) or interactions with other elements. If the claims are substantiated, incorporating macroalgae into rabbit feed could serve as part of a market strategy to appeal to consumers concerned about meat quality and promote a sustainable circular economy.

## 2. Results and Discussion

### 2.1. Macrominerals

**Figure 1:**
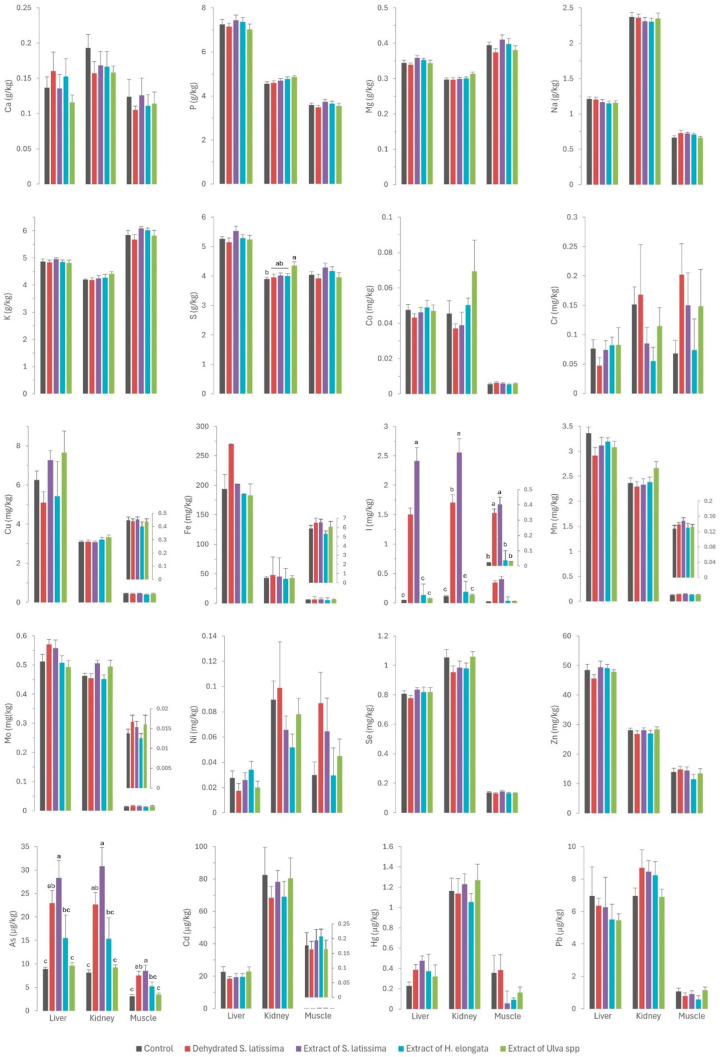
Contents of macrominerals (Ca—calcium; P—phosphorus; Mg—magnesium; Na—sodium; K—potassium; S—sulfur; expressed in g/kg wet weight), microminerals (Co—cobalt; Cr—chromium; Cu—copper; Fe—iron; I—iodine; Mn—manganese; Mo—molybdenum; Ni—nickel; Se—selenium; Zn—zinc; expressed in mg/kg wet weight), and toxic elements (As—arsenic; Cd—cadmium; Hg—mercury; Pb—lead; expressed in µg/kg wet weight) in liver, kidney, and muscle of rabbits fed with the different macroalgae diets. Results are expressed as mean ± SEM. Different letters indicate statistically significant differences between groups.

**Figure 2:**
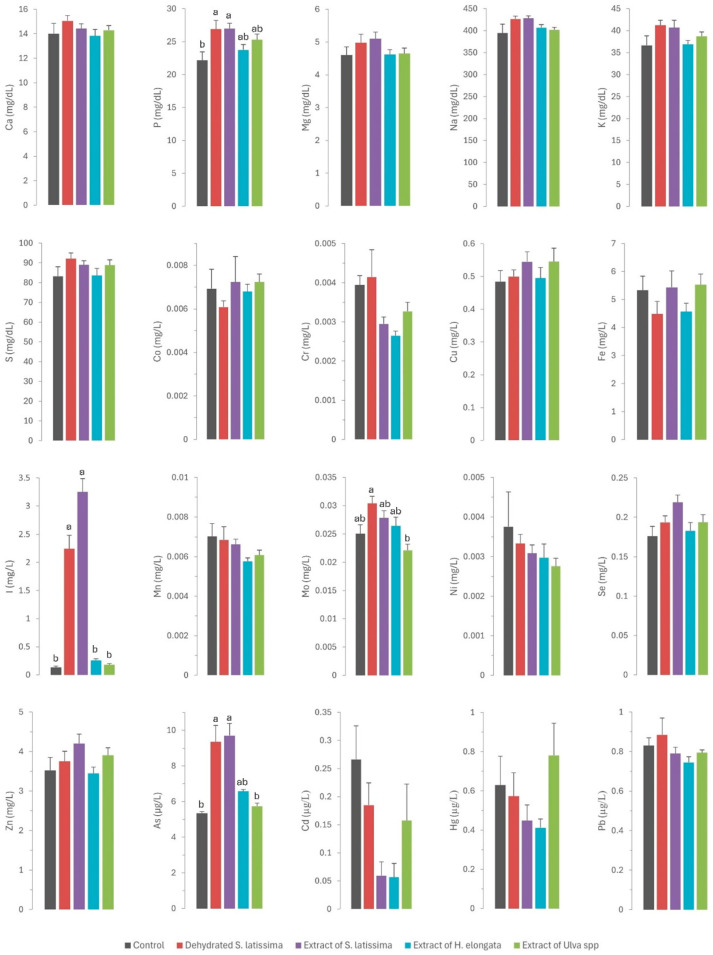
Contents of macrominerals (Ca—calcium; P—phosphorus; Mg—magnesium; Na—sodium; K—potassium; S—sulfur; expressed in mg/dL), microminerals (Co—cobalt; Cr—chromium; Cu—copper; Fe—iron; I—iodine; Mn—manganese; Mo—molybdenum; Ni—nickel; Se—selenium; Zn—zinc; expressed in mg/L), and toxic elements (As—arsenic; Cd—cadmium; Hg—mercury; Pb—lead; expressed in µg/L) in serum of rabbits fed the different macroalgae-supplemented diets. Results are expressed as mean ± SEM. Different letters indicate statistically significant differences between groups.

The results of the mineral profile analyses of the tissues (liver, kidney, and muscle) and serum in rabbits fed the different diets supplemented with macroalgae are presented in Figure 1 and Figure 2, respectively.

Concentrations of macrominerals in the control group were within the previously reported ranges [1,27,28]. Rabbit meat is characterized by very low amounts of sodium and very high contents of potassium and phosphorus relative to other meats such as pork, beef, and chicken (ranging between 0.6 and 1.0 g sodium/kg meat, 1.5 and 3.7 g potassium/kg meat, and 1.5 and 2.2 g phosphorus/kg meat [27,29]). These characteristics make rabbit meat particularly recommended for diets aimed at preventing hypertension [29]. 

The elevated concentration of macrominerals in some of the macroalgae species included in our study (Table 1) had minimal impact on both serum and tissue macromineral concentrations. These findings are consistent with expectations because the control diet was formulated to include the recommended concentrations of each macromineral. The absence of discernible differences among the experimental groups indicates proficient homeostatic regulation of macrominerals [30] once physiological needs are met.

The *Ulva* spp. extract (EU) emerged as the only treatment that had a major impact on macromineral tissue deposition. This extract had an exceptionally high sulfur concentration (117 g/kg DM), exceeding those in the other macroalgae products fed by one order of magnitude. This resulted in a 26.2% increase in sulfur concentration in the diet. This led to increased kidney sulfur concentrations (4.36 g/kg) compared to the control group (3.89 g/kg) and the groups supplemented with other macroalgae. These findings may be attributed to the renal excretion of this mineral, as previously reported [30]. The lack of effects on other tissues can be considered a positive outcome. Nevertheless, sulfur is known to be an antagonist of selenium [24], mainly in ruminants [25,26,31], as both elements show similar chemical and physical properties [26]. In these studies, high concentrations of sulfur in the diet reduced the concentration of selenium in the liver and plasma, which would have a negative effect in selenium-deficient areas [25]. Therefore, studying the effect of the high content of sulfur in some macroalgae on the selenium content in muscle, liver, and kidney is of interest. 

Moreover, the *Ulva* spp. extract was rich in magnesium (43.5 g/kg DM), with values one order of magnitude higher than in other macroalgae products, resulting in a 23.8% increase in the diet. While there was a tendency for kidney magnesium concentrations (0.313 g/kg) to be higher (*p* = 0.101) than in the control (0.296 g/kg) and other macroalgae-supplemented groups, no other differences were observed in tissue magnesium concentrations. This suggests a potential role of the kidney in the homeostatic regulation of this macromineral, mirroring the pattern observed for sulfur.

The sodium content of all the macroalgae products examined in the study was rather high, ranging from 26.9 to 99.9 g/kg DM. This represents an increase of up to 28.7% in dietary sodium content relative to that supplied by the basal diet. Despite these considerable variations, no statistically significant differences were detected across the experimental groups. This outcome is particularly encouraging as the high salt content has been a matter of concern for the use of some macroalgae in livestock diets, especially in the context of rabbit meat, which is valued for its low sodium content. The latter attribute makes rabbit meat highly recommended for individuals following diets aimed at preventing hypertension [27].

The statistically significant increase (*p* = 0.011) in serum phosphorus concentrations (Figure 2) across all experimental groups (ranging from 24.3 to 26.9 mg/dL) relative to the control group (22.1 mg/dL), especially in both *S. latissima* groups, is surprising. This finding was unexpected given the very low phosphorus concentrations in all macroalgae tested (ranging from 1.67 to 4.17 g/kg DM) relative to the basal control diet (7.18 g/kg DM). Although the values are consistent with physiological concentrations, they may indicate higher bioavailability of phosphorus in macroalgae than in other feed ingredients. Numerous authors have observed a low phytic acid content in many macroalgae species below those in most feed ingredients, which indicates that the bioavailability of phosphorus provided by macroalgae would be quite high. Therefore, macroalgae may be a valuable source of this element [10,11,32]. Such positive outcomes are noteworthy as reducing phosphorus in livestock diets is of great environmental concern [33]. This aspect warrants further research.

### 2.2. Microminerals

The concentrations of microminerals in tissues and serum were within the usual ranges previously reported in the literature for rabbits [1,27,28]. Rabbit meat, like other types of white meat, is characterized by a lower iron content than red meat (ranging between 18 and 46 mg Fe/kg), while the selenium content is higher than in other meats like veal (<0.10 mg selenium/kg meat) but similar to that in beef or chicken (0.15–0.17 mg selenium/kg meat) [29,34]. 

Iodine was the only element for which there were significant differences between experimental groups (Figure 1). The iodine content was very high (up to 4 orders of magnitude) in *S. lattisima,* both in the dehydrated form (4446 mg/kg DM) and the extract (6627 mg/kg DM), and to a lesser extent in the *H. elongata* extract (220 mg/kg DM) and the *Ulva* spp. extract (36 mg/mg DM), which resulted in an increase of between 45 and 8099% in the iodine concentrations in the experimental diets (Table 1). The high concentrations of iodine in the experimental diets resulted in a very high iodine absorption in the gut (not measured directly but inferred from the serum iodine concentrations: 2.24 and 2.79 mg/L in SL and ESL groups, and 0.715 and 0.179 mg/L in EHE and EU groups, compared to 0.133 mg/L in the control group). Iodine is regulated by renal homeostasis; unlike most macro and microminerals, iodine is absorbed in excess in the gut, and the excess is excreted in the kidney or, in the case of hens and female mammals, in the eggs or milk [12]. The homeostatic control, which also applies to selenium, allows the production of iodine-enriched animal products [13]. The iodine concentration in the muscle was significantly higher in the SL, ESL, and EHE groups (respectively 17, 20, and 2 times higher) than in the control group. It was also higher in the liver (respectively, 32, 52, and 3 times higher) and kidney (respectively, 15, 23, and 2 times) of the three experimental groups than in the control group. These results confirm that macroalgae are a good dietary source of iodine, which is successfully transferred to animal tissues due to the weakness of the links between polysaccharides and iodine [9]. The findings also indicate that iodine provided by macroalgae is more bioavailable than inorganic iodine, as previously reported [12,13,35].

**Figure 3:**
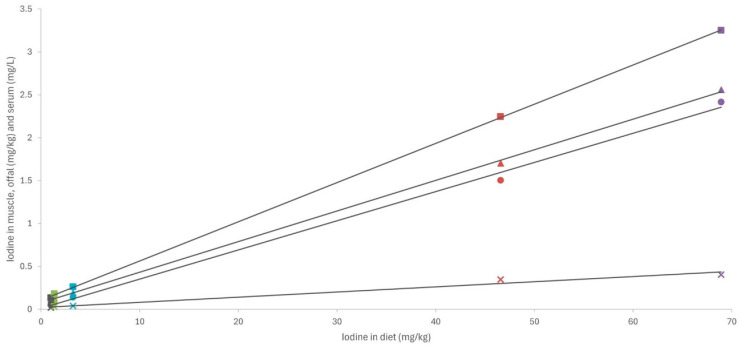
Linear relationship between iodine content in diet (mg/kg dry matter) and in muscle, liver, and kidney (mg/kg wet weight) and serum (mg/L). SL—dehydrated *S. latissima*; ESL—extract of *S. latissima*; EHE—extract of *H. elongata*; EU—extract of *Ulva* spp.

**Figure 4:**
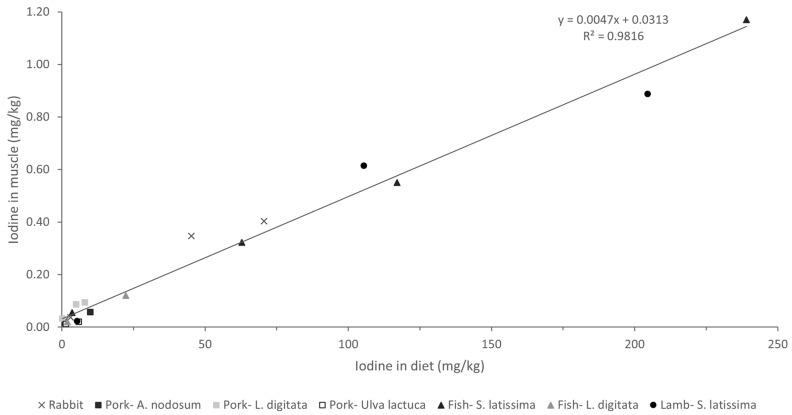
Iodine deposition in muscle (mg/kg wet weight) of animals fed different macroalgae-supplemented diets related to iodine content in diet (mg/kg dry weight). Values refer to the present study (rabbit) and to studies carried out with different macroalgae in porcine (*Ascophyllum nodosum* [13], *Laminaria digitata* [35], *Ulva lactuca* [36]), fish (*S. latissima* [22], *L. digitata* [37]), and lamb (*S. latissima* [38]).

Analysis of the association between iodine concentrations in different diets and tissues revealed a strong linear relationship (Figure 3; R > 0.98; *p* < 0.001). These findings are particularly interesting as they suggest a consistent pattern of iodine bioavailability and tissue deposition across all macroalgae species and/or products tested. Moreover, a comparison of our results with those of other studies concerning different animal species (including fish) fed various types of macroalgae in their diets, with a wide range of inclusion (up to 240 mg iodine/kg complete feed), revealed a very similar pattern of iodine deposition in muscle (Figure 4; R = 0.98; *p* < 0.001). This uniform response suggests the possibility that iodine concentrations in meat and meat products could be easily adapted to specific population needs. While iodine is crucial for maintaining gut health and productivity, excessive concentrations can have deleterious effects in both humans and animals, necessitating careful regulation within appropriate ranges [13]. 

Most meats are generally characterized by a low iodine content, typically ranging from 0.02 to 0.06 mg/kg. By contrast, other food items, such as milk (0.10 mg/kg), cheese (0.20 mg/kg), eggs (0.33 mg/kg), and fish (0.13 mg/kg), are much richer in this essential element [13]. However, taking into consideration the iodine concentrations allowed by the European legislation (10 mg iodine/kg complete feed), supplementation of the rabbit diets with brown macroalgae yielded high iodine concentrations in the muscle (0.35–0.40 mg/kg), equivalent to those produced by some of the iodine-rich foods mentioned earlier. Several macroalgae species have been identified as of potential value for enhancing the iodine content of meat and other animal-derived products, thereby contributing to mitigating iodine deficiency, which is endemic in many parts of the world [13]. Previous studies have successfully enriched meat with iodine by incorporating specific macroalgae species into livestock diets (Figure 4). Moreover, macroalgae have been suggested to be suitable for supplying iodine in organic livestock productions, in which mineral supplementation is more limited. For instance, in dairy cows, a supplement composed of *Ulva rigida, Sargasum muticum*, and *Saccorhiza polyschides* (administered at 100 g/animal/day) doubled the iodine content in milk, effectively addressing the animals’ iodine deficiency and resulting in iodine-enriched milk [17]. 

With the exception of iodine, the concentrations of most other trace minerals were consistently low in all the macroalgae products tested in our study relative to the basal/control diet (Table 1), indicating that these macroalgae do not provide any additional minerals. Consequently, no effect of algae supplementation on the micromineral content of the muscle and viscera was expected. Nevertheless, the iron content in the liver tended to be higher (*p* = 0.085) in the SL group (269 mg/kg) than in the control (193 mg/kg) and the other experimental groups (182–202 mg/kg). Considering that the iron concentration of SL, although higher than in other seaweeds, is lower than that provided by the basal diet, these results may suggest higher bioavailability of the iron provided by this macroalgae than by other feed ingredients. Some authors have already reported that iron bioavailability is about 92.4% in *Laminaria* spp. [39], and that there is a positive correlation between soluble fiber (constituted by some carbohydrates found in macroalgae like laminarin or fucoidan) and the bioavailability of some minerals. Moreover, the low phytic acid content in macroalgae may enhance the bioavailability of other minerals like iron, as previously reported for phosphorus, as phytic acid can act as an anti-nutritive factor for some elements [10,11]. 

A similar pattern to that described for iron was observed for molybdenum. Thus, the inclusion of SL in the diet, with a higher molybdenum content than the other macroalgae but lower than the control diet, led to a higher molybdenum concentration in serum (*p* = 0.002) and a tendency in the liver (*p* = 0.088) relative to the control and other macroalgae groups. However, there is no information in the scientific literature regarding the bioavailability of molybdenum in algae. Another possible explanation is the existence of an interaction with other elements in the diet. A previous study by our group in dairy cows fed macroalgae-supplemented diets also showed a significant effect on serum molybdenum concentrations and milk excretion, suggesting an effect of the sulfur content of the macroalgae supplement on this element [17]. Another possible explanation is the interaction between molybdenum and other elements that are excreted by the kidneys, such as iodine [30]. In addition to the already mentioned interaction between sulfur and selenium, remarkable interactions between iodine and arsenic and selenium have already been reported: high concentrations of both iodine and arsenic can decrease selenium concentration [21,22,23], whereas high contents of selenium can mitigate certain toxic elements like arsenic [40]. This interaction often requires attention in practice to prevent the consequences of competitive interactions at high concentrations on selenium status in animals. Selenium has numerous functions in the body as an essential component of antioxidant enzymes, and it has a key role in shielding cells from oxidative damage and bolstering immune function, which is critical for maintaining gut health [41]. In the present study, the selenium concentration was consistent across all diets, and no differences were observed between the experimental groups in regard to selenium concentrations in serum and tissues (see Figure 1 and Figure 2). These findings are noteworthy, as they allow the unlimited inclusion of these macroalgae in animal diets.

### 2.3. Toxic Elements

Residues of toxic elements in the serum and tissues of rabbits fed the control diet were remarkably low, indicative of minimal environmental exposure through the diet., The concentrations of toxic elements in the control diet were actually much lower (Table 1) than in other livestock diets (arsenic, 0.31–0.85 mg/kg; cadmium, 0.07–0.19 mg/kg; mercury, 0.012–0.05 mg/kg; lead, 0.07–1.16 mg/kg), and especially fish diets (arsenic, 4.25 mg/kg; cadmium, 0.17 mg/kg; mercury, 0.06 mg/kg; lead, 0.07 mg/kg; [42]). Both arsenic and mercury reach very high values in these diets as they contain large amounts of fish meal, which is very rich in these toxic elements [43]. In our study, the concentrations of toxic element residues found in muscle and liver were similar or even lower than previously reported [44]. Nevertheless, when considered together with toxic element concentrations in the liver and kidney of other livestock species in Spain [45,46], the concentrations in rabbits were found to be remarkably lower. This discrepancy can probably be attributed to the relatively short lifespan of rabbits at the time of slaughter, especially in comparison to other farm species, notably ruminants.

**Figure 5:**
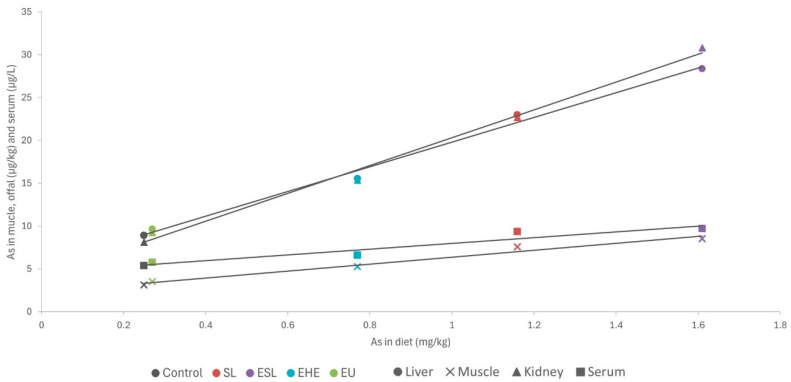
Linear relationship between arsenic content in diet (mg/kg dry matter) and in muscle, liver, and kidney (µg/kg wet weight) and serum (µg/L). SL—dehydrated *S. latissima*; ESL—extract of *S. latissima*; EHE—extract of *H. elongata*; EU—extract of *Ulva* spp.

Except for arsenic, the concentrations of toxic elements in the macroalgae were very low (<0.5 mg/kg DM) and similar to those in the control diet. Consequently, no differences were observed in the residues of cadmium, lead, and mercury in the serum and tissues of rabbits (as shown in Figure 1 and Figure 2). By contrast, in a pattern similar to that of iodine, arsenic concentrations in macroalgae were very high, reaching up to three orders of magnitude higher than in the control diet. This increase was notable in *S. latissima*, both in the dehydrated form (89.3 mg/kg DM) and in the extract (133 mg/kg DM), and to a lesser extent in the extracts of *H. elongata* (51.9 mg/kg DM) and *Ulva* spp. (2.25 mg/mg DM). Consequently, arsenic concentrations in the experimental diets increased to between 7.8% and a remarkable 558.4%. The elevated arsenic concentrations in the experimental diets translated into significantly higher concentrations of arsenic residues in both serum and tissues than in the control group (Figure 1 and Figure 2), with a robust linear correlation with the arsenic concentrations in the diet (see Figure 5; R^2^ > 0.93; *p* < 0.001). Although higher than in the control, arsenic residues in the liver and kidneys (reaching up to 30 µg/kg, representing a three-fold increase relative to the control group) and notably in the muscle (reaching up to 8.5 µg/kg, approximately three times that in the control group) were relatively low in comparison with concentrations commonly found in meat, liver and kidney [42], and much lower than in other foods [47] such as rice (90–150 µg/kg), and similar to other foods such as eggs (8 µg/kg) or milk (3 µg/kg). In any case, arsenic content was found to be well below the maximum permitted concentrations for other foods (Regulation EU 915/2023).

Furthermore, the macroalgal species included in these experimental diets have been shown to contain very low concentrations of inorganic arsenic (0.15–0.59 mg/kg DM), the most toxic form of arsenic. Most of the arsenic in macroalgae exists in organic forms of minimal toxicity [20]. Transfer of low quantities of inorganic arsenic in animal tissues is also minimal, resulting in meat making an insignificant contribution to human exposure to arsenic [42,47]. Additionally, arsenic has been found to be less bioavailable in macroalgae than in fishmeal or fish oil, which are primary sources of arsenic in animal feed [22,30]; consequently, the deposition of arsenic in meat is limited.

## 3. Materials and Methods

### 3.1. Experimental Trial

This study was conducted within a broader experimental trial (TIRAC project) aimed at evaluating the benefits of four marine macroalgae products on the gut health of post-weaning rabbits. As part of this research, the effects of macroalgae supplementation on the mineral composition of muscle, liver, kidney, and serum of rabbits were assessed. All of the experimental procedures were in accordance with the Spanish guidelines for the care and use of animals in research (Spanish Royal Degree 53/2013; BOE, 2013). The procedures were approved by the Bioethics Committee of the Universidad Politécnica de Madrid, Spain (protocol code 2021–002, approved 24 February 2021). 

In this trial, experimental diets, each including one of four macroalgae products, were tested. The macroalgae products were selected according to the results of their in vitro characterization [48] and the availability of discards from industrial processing. Based on these criteria, *Saccharina latissima* (sugar kelp) was tested in two formats: dehydrated macroalgae (SL) and an aqueous extract of the hydrolyzed macroalgae (ESL); *Himanthalia elongata* (sea spaghetti) was tested as an aqueous extract (EHE), and *Ulva* spp. (sea lettuce) was tested in the form of the aqueous extract of the hydrolyzed macroalgae (EU). The products were provided by Porto-Muíños S.L. (Cerceda, A Coruña, Spain) and elaborated from discards generated during industrial processing, as previously described [48]. The basal growing diet (Table 2), formulated according to the nutrition standards for growing rabbits, was supplemented with macroalgae products at a concentration of 1.025% (dry matter). The rabbits did not receive any antibiotics throughout the trial. 

A total of 40 crossbred, mixed-sex (New Zealand White X Californian), weaned rabbits were haphazardly assigned to each experimental group and to a control group. Each group was composed of 8 rabbits, which were fed *ad libitum* with the corresponding experimental diet between weaning (30 days of age, 701 ± 80 g) to slaughter (63 days of age, 2154 ± 162 g). Each rabbit was housed individually in a flat desk cage and had *ad libitum* access to water. Overall, the rabbits receiving the macroalgae treatment were in good health, and no statistically significant differences compared to the control group were found in terms of mortality and performance.

### 3.2. Sample Collection

At age 63 days, the rabbits were slaughtered by head concussion. Before the rabbits were bled, a sample of fresh blood (10 mL) was collected by cardiac puncture. The blood was then centrifuged to obtain a serum sample. The liver, kidneys, and one of the thighs were collected from each carcass, packed in plastic bags, and immediately transported to the laboratory. Visible fat, connective tissue, and major blood vessels were removed from the organs, which were then homogenized and stored at −18 °C and processed within one month for analysis.

### 3.3. Sample Preparation

For the determination of iodine content by inductively coupled plasma mass spectrometry (ICP-MS), the European Standard guideline [50] was applied. Briefly, 1 mL serum or 0.2 g tissue (liver, kidney, or muscle) was placed in a 15 mL Falcon tube, followed by the addition of 0.4 mL of tetramethylammonium hydroxide solution (TMAH, Sigma Aldrich, Merck, Darmstadt, Germany) and 2 mL of ultrapure water. The mixture was then thoroughly mixed and incubated at 90 °C for 3 h in a preheated drying oven. The mixture was cooled, and ultrapure water was added to a final volume of 5 mL. The tubes were then centrifuged at 4000 rpm for 15 min to remove coarse particles. Subsequently, the supernatant was filtered through a 0.45 µm filter into a 15 mL tube. The prepared samples were measured within one day.

For determination of the other trace elements, samples were acid digested. For processing serum samples, 1 mL of each sample was mixed with 0.6 mL of concentrated (69%) nitric acid (TMA, Hiperpure, PanReac, Barcelona, Spain) and 0.4 mL of H_2_O_2_ (PanReac, Barcelona, Spain). The mixture was digested for 24 h in a preheated drying oven at 60 °C, and ultrapure water was then added to a final volume of 5 mL, and tubes were centrifuged at 3000 rpm for 5 min. The supernatant was transferred to another tube for analysis. For muscle, kidney, and liver, mineralization was carried out in a microwave-assisted digestion system (240 °C, 40 bar; Ethos Plus, Milestone, Sorisole, Italy). One g of sample was mixed with 3 mL of concentrated (69%) nitric acid and 1 mL of ultrapure water. Once completely digested, the samples were transferred to polypropylene tubes, and ultrapure water was added to a final volume of 50 mL. 

### 3.4. Mineral Analysis

The concentrations of macrominerals (Ca, P, Mg, Na, K, and S), essential trace elements (Co, Cr, Cu, Fe, I, Mn, Mo, Ni, Se, and Zn) and potentially toxic trace elements (As, Cd, Hg, and Pb) in all the digested samples were determined by ICP-MS (Agilent 7900×ICP-MS system; Agilent Technologies, Tokyo, Japan). The samples were analyzed in triplicate, and analytical quality control was performed throughout the process. Blanks were included in each batch and measured between samples. Limits of detection (LD) were calculated as three times the standard deviation of the blanks, and they were found to be sufficient for the determination of the concentrations of minerals in the samples in all cases. Replicates of two certified reference materials (fish muscle, ERM-BB422, and bovine liver, BCR-185R: Institute for Reference Materials and Measurements, Geel, Belgium) were analyzed following the same procedure as for the experimental samples to evaluate the accuracy of the analytical method (Table 3). In the case of minerals not certified by the CRM (Cr, Co, P, Ni, Mo, and S), spiked samples were used at concentrations producing absorbance values up to 10 times the usual concentrations in muscle. The analytical recoveries of these elements ranged between 96 and 108%. The results obtained for the reference materials and the spiked samples showed satisfactory recoveries between the measured and certified values (recovery between 75 and 125% is considered admissible); therefore, the accuracy of the determinations was acceptable.

### 3.5. Statistical Analysis

Distinct X_40×20_ matrices were constructed for rabbit tissues (blood, kidney, liver, and muscle), including samples from the 4 treatments plus the control, with 20 elements per row. The effect of macroalgae treatments on the mineral composition of each sample type was assessed through a Kruskal–Wallis test followed by a post-hoc Tukey–Kramer test at a significance level of α = 0.05. Linear regression analysis was conducted to assess the associations between the mineral profile of diets and tissue by determining the coefficient of determination R^2^.

## 4. Conclusions

In summary, the study findings demonstrate that supplementing rabbit diets with 1.025% macroalgae (dry matter) to improve gut health leads to remarkable changes in iodine and arsenic concentrations in the muscle, liver, and kidney. While the notable increase in iodine concentration, dependent on dosage, is promising, particularly in regions with low iodine endemicity, elevated arsenic residues above control concentrations may be of concern. However, arsenic residue concentrations in rabbits fed macroalgae-supplemented diets remained relatively low, similar to those found in conventionally fed animal meats, and much lower than in other food sources. Moreover, the absence of interactions with other micronutrients, particularly selenium, suggests that incorporating macroalgae in animal diets at the concentrations tested does not restrict the mineral content. The apparent enhanced bioavailability of elements such as phosphorus and iron, as indicated by our findings, warrants further research as it could yield beneficial effects and should be considered in diet formulation to potentially reduce the need for mineral supplements and mitigate environmental impacts, especially regarding phosphorus utilization.

## Figures and Tables

**Table 1:** Mineral composition of the macroalgae products studied and complete feed. For the experimental diets (SL, ESL, EHE, EU), 1.025% of each macroalgae product was additionally added to the corresponding control diet. SL—dehydrated *S. latissima*; ESL—extract of *S. latissima*; EHE—extract of *H. elongata*; EU—extract of *Ulva* spp.

		Macroalgae				Complete Feed												
	SL	ESL	EHE	EU		Control		SL			ESL			EHE			EU	
**Macrominerals (g/kg)**																		
Ca	20.8	8.13	10.5	9.35		11.7		11.4	(−2.6) *		11.6	(−0.9)		11.4	(−2.6)		11.6	(−0.9)
P	2.8	4.17	1.67	2.25		7.18		6.99	(−2.6)		6.81	(−5.3)		6.69	(−6.8)		7.23	(0.7)
Mg	6.79	7.98	9.37	43.5		2.98		3.05	(2.3)		2.87	(−3.7)		3.07	(3.0)		3.69	(23.8)
K	144	45.2	231	13.6		13.2		14.9	(12.9)		13.6	(3.0)		15.5	(17.4)		14.5	(9.8)
Na	54	30.6	99.9	26.9		3.49		4.29	(22.9)		3.69	(5.7)		4.49	(28.7)		3.82	(9.5)
S	10.8	13.2	25.6	117		4.39		4.69	(6.8)		4.57	(4.1)		4.84	(10.3)		5.54	(26.2)
**Microminerals (mg/kg)**																		
Co	0.12	0.07	0.86	0.14		0.583		0.621	(6.5)		0.574	(−1.5)		0.571	(−2.1)		0.59	(1.2)
Cr	3.32	1.33	2.67	0.84		5.69		5.46	(−4.0)		5.77	(1.4)		5.66	(−0.5)		5.64	(−0.9)
Cu	0.85	0.38	0.87	0.62		24.1		23.6	(−2.1)		25.3	(5.0)		24.7	(2.5)		23.4	(−2.9)
Fe	281	85.6	33.1	31.9		469		460	(−1.9)		447	(−4.7)		438	(−6.6)		423	(−9.8)
I	4446	6627	220	36		0.861		45.2	(5149)		70.6	(8099)		2.99	(247)		1.25	(45.2)
Mn	8.18	5.29	49.4	17.2		67.9		65.9	(−2.9)		69.1	(1.8)		71.1	(4.7)		70	(3.1)
Mo	0.34	0.29	0.17	0.18		1.35		1.25	(−7.4)		1.27	(−5.9)		1.42	(5.2)		1.38	(2.2)
Ni	0.82	1.18	5.88	2.27		3.15		3.22	(2.2)		3.33	(5.7)		3.08	(−2.2)		3.19	(1.3)
Se	0.14	0.07	0.05	0.06		0.478		0.455	(−4.8)		0.474	(−0.8)		0.464	(−2.9)		0.46	(−4.2)
Zn	25.9	49.2	9.67	6.08		115		115	(0.0)		120	(4.3)		107	(−7.0)		115	(0.0)
**Toxic elements (mg/kg)**																		
As	89.4	133	51.9	2.25		0.245		1.151	(369.8)		1.613	(558.4)		0.772	(215.1)		0.26	(7.8)
Cd	0.34	0.15	0.53	0.04		0.114		0.119	(4.4)		0.108	(−5.3)		0.121	(6.1)		0.12	(3.5)
Hg	0.019	0.009	0.003	0.001		0.016		0.016	(1.9)		0.017	(7.5)		0.016	(4.4)		0.02	(3.1)
Pb	0.49	0.82	0.39	0.07		0.201		0.217	(8.0)		0.202	(0.5)		0.211	(5.0)		0.21	(6.5)

* in parentheses is the percentage of variation relative to the control diet.

**Table 2:** Formulation and chemical composition of the control diet.

Ingredient	Content (%)	Chemical Composition	Content (%)
Alfalfa meal	20.00	Dry matter	88.90
Wheat bran 15% CP	19.98	Ash	7.23
Orange pulp	15.00	Crude protein	14.40
Sunflower meal extracted	11.82	Crude fat	2.50
Oat hulls	10.00	Crude fiber	17.19
Corn gluten feed	8.00	NDF	37.90
Barley	4.54	ADF	20.47
Beet molasses	3.00	ADL	5.43
Soybean meal 47% CP	2.81	Lysine	0.65
Rice hulls	2.17	Methionine	0.23
Corn DDGS	1.04	Met + Cys	0.47
Soybean oil	0.50	Threonine	0.59
Sodium Chloride	0.39	Arginine	0.83
Vitamin and mineral premix ^1^	0.30	**Nutritive value ^2^**	
Diclazuril 0.5%	0.20	Digestible energy, kcal	2150.00
L-Lysine sulphate	0.16	Metabolizable energy, kcal	2169.36
L-Threonine	0.09	Digestible protein, %	10.39

^1^ The vitamin and mineral premix provided the following components per kg of complete diet: Vit A, 10,000 IU; Vit D_3_, 1000 IU; Vit E, 40 mg; Vit K_3_, 2 mg; Vit B_1_, 2 mg; Vit B_2_, 6 mg; Vit B_6_, 3.9 mg; Vit B_12_, 15 µg; calcium pantothenate, 16.30 mg; niacin, 40 mg; biotin, 100 µg; folic acid, 1 mg; betaine hydrochloride, 131.6 mg; Mn, 10 mg; I, 1 mg; Fe, 50 mg; Cu, 8 mg; Zn, 50 mg; Co, 0.2 mg; Se, 0.2 mg. ^2^ [49].

**Table 3:** Limits of detection (µg/L) of each element, certified concentrations (mg/kg), % of recovery of the CRM, and % of recovery of spiked samples.

	LD	Fish Muscle ERM-BB422		Bovine Liver BCR-185R		Spiked Samples
		Certified Concentrations	% Recovery	Certified Concentrations	% Recovery	% Recovery
**Ca**	0.30	410	119	-	-	103
**P**	0.008	-	-	-	-	96
**Mg**	0.31	1566	114	-	-	94
**Na**	1.25	3507	125	-	-	93
**K**	7.37	26,835	120	-	-	91
**S**	0.61	-	-	-	-	105
**Co**	0.005	-	-	-	-	101
**Cr**	0.03	-	-	-	-	108
**Cu**	0.013	1.67	105	277	92	107
**Fe**	0.19	9.40	107	-		107
**I**	0.165	1.4	117	-	-	103
**Mn**	0.02	0.368	105	11.07	105	114
**Mo**	0.006	-	-	-	-	106
**Ni**	0.014	-	-	-	-	104
**Se**	0.005	1.33	99	1.68	91	114
**Zn**	0.18	16.00	110	138.6	109	107
**As**	0.006	12.7	114	0.033	100	102
**Cd**	0.008	0.0075	99	0.544	91	100
**Hg**	0.063	0.601	96	-	-	94
**Pb**	0.002	-	-	0.172	95	96

## Data Availability

Data are contained within the article.
